# Modeling the potential impact on the US blood supply of transfusing critically ill patients with fresher stored red blood cells

**DOI:** 10.1371/journal.pone.0174033

**Published:** 2017-03-20

**Authors:** Arianna Simonetti, Hussein Ezzeldin, Mikhail Menis, Stephen McKean, Hector Izurieta, Steven A. Anderson, Richard A. Forshee

**Affiliations:** 1 Office of Biostatistics and Epidemiology, Center for Biologics Evaluation and Research, US Food and Drug Administration, Silver Spring, MD, United States of America; 2 Acumen LLC, Burlingame, CA, United States of America; Massachusetts Institute of Technology, UNITED STATES

## Abstract

**Background:**

Although some studies have suggested that transfusion recipients may have better medical outcomes if transfused with red blood cell units stored for a short time, the overall body of evidence shows mixed results. It is important to understand how using fresher stored red blood cell units for certain patient groups may affect blood availability.

**Methods:**

Based on the Stock-and-Flow simulation model of the US blood supply developed by Simonetti et al. 2014, we evaluated a newly implemented allocation method of preferentially transfusing fresher stored red blood cell units to a subset of high-risk group of critically ill patients and its potential impact on supply.

**Results:**

Simulation results showed that, depending on the scenario, the US blood total supply might be reduced between 2-42%, when compared to the standard of care in transfusion medicine practice. Among our simulated scenarios, we observed that the number of expired red blood cell units modulated the supply levels. The age threshold of the required red blood cell units was inversely correlated with both the supply levels and the number of transfused units that failed to meet that age threshold.

**Conclusion:**

To our knowledge, this study represents the first attempt to develop a comprehensive framework to evaluate the impact of preferentially transfusing fresher stored red blood cells to the higher-risk critically ill patients on supply. Model results show the difficulties to identify an optimal scenario.

## Introduction

Observational studies have estimated the effects of red blood cell (RBC) storage time on clinical outcomes among different patient groups. Some studies have shown that transfusing certain high-risk patients, such as cardiac surgery patients, with RBC units that have been stored for prolonged time may increase the risk of adverse outcomes [1–6]. Other studies have shown no difference between those receiving fresher stored RBC units compared to those transfused using the standard of care [7, 8].

Results from randomized clinical trials (RCT) have varied in their outcomes. The RECESS RCT from the National Heart, Lung, and Blood Institute [7] found no difference in serious adverse events or mortality among groups of cardiac surgery patients transfused with older stored RBCs. A study presented in the Canadian Cardiovascular Congress 2014 [8] followed 2,015 cardiac surgery patients over nine years (2005-2013) at the New Brunswick Heart Centre demonstrates that the group transfused with fresher stored RBCs had better results in all outcomes (i.e., mortality, infection, renal failure, mean length of stay). The Age of Blood Evaluation (ABLE) study [9] describes a multicenter randomized, blinded trial in critically ill adults. Patients enrolled from tertiary intensive care units were transfused with RBCs stored for either less than 8 days (1,211 patients) or the oldest compatible RBCs available in the blood bank (1,219). The study, which used a primary outcome measure of 90-day all-cause mortality, did not find statistically significant outcome differences between the two study groups. Results from these studies are probably not generalizable, given that critically ill patients at higher risk of adverse consequences due to prolonged red-cell storage were not included. Results from other ongoing clinical trials may help clarify the relationship between the age of transfused RBCs and patient outcomes. The results of these studies could affect blood transfusion practices if fresher stored RBCs are shown to improve patient outcomes. If there were changes in transfusion practice, it would be important to evaluate how using fresher stored RBCs may potentially impact the US blood supply. The US blood supply would be reduced if the shelf-life of RBCs was shortened from a maximum of 42 days (current policy). As an alternative, selected groups of high-risk patients could be preferentially transfused with fresher stored red cells potentially resulting in beneficial effects on the overall patients’ survival without significantly affecting the US overall blood supply.

Based on the Stock-and-Flow simulation model of the US blood supply developed by Simonetti et al. 2014 [10], we explored the potential impact on the US blood supply of transfusing fresher stored RBCs to a subset of pre-defined critically ill patients (high-risk groups) as opposed to the baseline recipient (low-risk group). We reviewed the literature to identify groups of patients that included the critically ill, such as trauma, cardiac surgery, intensive care unit and coronary care unit patients, who are potentially at higher risk of adverse events if transfused with older RBCs [1–6]. We extracted information on RBC units transfused from the Centers for Medicare and Medicaid Services (CMS) [11, 12]. Next, we implemented a new allocation method for modeling preferential transfusion practices with fresher stored RBCs for patients belonging to a high-risk group. To understand the potential impact on the US blood supply, we evaluated simulated scenarios (a) for baseline, assuming that current policies are maintained and (b) for high-risk groups, assuming they are preferentially transfused with fresher stored RBCs. We developed specific assumptions for the age of RBCs to transfuse to each high-risk group from a literature review [1–6].

## Materials and methods

### A new allocation method to distribute blood: The ‘Threshold Method’

The US blood supply model developed by Simonetti et al. [10] evaluates blood supply levels under three different allocation methods: First-In-First-Out (FIFO), Likely Oldest (LO), and Likely Newest (LN). In this study, we implemented a ‘Threshold Method’ [13] (TM) to distribute RBC units of a certain age to patients in specific high-risk groups. We divided blood recipients into two groups. The High Risk Groups (HRGs) included critically ill patients who were potentially at a higher risk for adverse events if they received a transfusion of older RBCs. The Baseline Blood Recipients (BBRs) are patients not belonging to the HRGs. The newly implemented algorithm affected the distribution of RBC units between the two model stocks of interest: the aggregate collector bank (Collector) and the aggregate blood distributor (Hospital) [10]. The TM algorithm attempted to allocate blood into the system: 1) from the Hospital to the HRGs recipients; and 2) from the Collector to the Hospital (S1 Fig). On each simulated day, the Hospital attempted to allocate blood to the HRGs in order to fulfill the average daily demand of RBCs from the specific group and the ‘threshold age’ of RBCs to be transfused. Then, for each blood type, the Hospital attempted to distribute RBC units of a specific age by first using an exact match. If no blood was available for cross matching, then the algorithm allocated compatible blood based on availability by age. If no compatible blood of a specific age was available to fulfill the HRGs needs, the algorithm provided the freshest stored RBC unit available using Last-In-First-Out. TM allocated compatible blood using the algorithm for phenotype compatibility rules [10] (S1 Table). The instances in which the Hospital failed to provide any RBC unit with the pre-specified age were recorded as ‘unmet RBC units by age.’ After allocating blood to the HRGs, the model attempted to allocate blood to the rest of the blood recipients. RBC units were distributed to BBRs using the LO allocation method [10].

The Collector attempted to supply the Hospital with enough young RBC units to meet the HRGs expected demand of blood for the next day. The Hospital received blood from the Collector to fulfill any need of blood demand regardless the RBCs age, while maintaining an inventory of 6 days of supply. Transfers of blood from the Collector to the Hospital occurred by using ‘standard’ phenotype compatibility rules (S2 Table). We defined the difference between the daily demand (requested RBC units) and the daily transfused as ‘unmet demand.’

The daily predictions of supply and demand were derived using aggregated blood donation information from America’s Blood Centers (ABC) [14], and updated blood utilization data among the inpatient US elderly (age 65+) billing information from the CMS for the period 2007-2012. We also used national blood collection and utilization estimates from the 2011 National Blood Collection and Utilization Survey [15] to reflect the 2010 US blood supply and estimated demand levels. We then generated quantitative estimates with uncertainty of the daily blood availability in the US for the overall blood and by ABO/Rh type [10]. We enhanced the phenotypic predictions to account for potential variability in the phenotype prevalence by using a Dirichlet-multinomial process (S3 Table, S2 and S3 Figs).

### Measures of performance of the blood system

We used the annual average daily (AAD) age of RBCs as a summary measure for the overall evaluation of the performance of the blood system for each simulated scenario. Specifically, we used the weighted AAD age of RBCs, which represented the distribution of RBC units among different blood ages. Other measures of performance were ‘unmet RBC units by age’ and ‘percentage of unmet RBC units by age’ for HGRs and BBRs. ‘Unmet RBC units by age’ represented the amount of RBC units that failed to meet the age requirement derived from the literature [1–6]. ‘Percentage of unmet RBC units by age’ was defined as the ratio between the number of RBC units that failed to meet the age requirement and the total number of units needed to meet that requirement. We used the percentage of met RBC units (100%—percentage of unmet RBC units) as a measure of the system efficiency. We also estimated the AAD total supply to represent the annual average daily number of RBC units available in the overall US blood supply system (collector + hospital), while the AAD expired RBC units was used to represent the annual average daily number of RBC units expired in the overall blood supply system (collector + hospital).

### High-risk groups’ identification and age of RBCs

We identified three HRGs—Cardiac Surgery (CS), Trauma (TR) and Intensive Care Unit (ICU) patients [2, 5]—from the literature [1–6]. Due to the different ward setting among hospitals, we also included Coronary Care Unit (CCU) patients. We used the International Classification of Diseases, 9th revision, Clinical modification (ICD-9-CM) codes [16] to identify patients belonging to the HRGs. We used Diagnosis codes to identify TR patients. We included fractures to the head and injuries of the abdomen, and extremities as trauma conditions that could potentially require blood transfusions [17–23]. We used the ICD-9-CM Procedure codes to identify CS patients who received RBC transfusion procedures (S4 and S5 Tables, SM). CCU and ICU patients were identified using Revenue codes, respectively. We ascertained blood utilization among the inpatient US elderly overall, annually, and for the identified HRGs for the years 2007-2012 by using the CMS databases. CMS data were used to ascertain average daily number of RBC units transfused, total number of RBC units transfused, and the percentage of total RBC units transfused for each of the mutually exclusive HRGs and their combinations among the inpatient US elderly during 2007-2012. Since a CS patient may also become an ICU patient if prognosis worsens, the same CS patient may be recorded in the database twice. In order to avoid double counting of patients and units transfused, we identified mutually exclusive groups of patients (Table 1). Table 1 illustrates the average percentage of total RBC units transfused and the corresponding 95% Confidence Interval for each of the mutually exclusive HRG and their combinations (S1 Fig).

**Table 1:** Average percent of total RBC units transfused for each of the mutually exclusive, one or more, of the High Risk Groups (HRGs) from CMS during 2007-2012.

**Mutually exclusive High Risk Groups (HRGs)**	**Average percent of total RBC units transfused, during 2007-2012**	**95% Confidence Interval**	
**One Group**		**Lower bound**	**Upper bound**
ICU Only	18.61%	18.59%	18.64%
CS Only	7.68%	7.63%	7.73%
CCU Only	4.72%	4.71%	4.73%
TR Only	2.09%	1.97%	2.21%
**Two Groups**			
CS ∩ ICU	16.43%	15.69%	17.13%
CS ∩ CCU	3.59%	3.58%	3.60%
ICU ∩ CCU	1.76%	1.73%	1.78%
TR ∩ ICU	1.23%	1.15%	1.31%
CS ∩ TR	0.33%	0.28%	0.37%
TR ∩ CCU	0.23%	0.21%	0.24%
**Three Groups**			
CS ∩ ICU ∩ CCU	3.85%	3.69%	4.01%
CS ∩ TR∩ ICU	1.03%	0.84%	1.22%
CS ∩ TR ∩ CCU	0.13%	0.11%	0.15%
TR ∩ ICU ∩ CCU	0.11%	0.10%	0.12%
**Four Groups**			
CS ∩ TR ∩ ICU ∩ CCU	0.18%	0.14%	0.22%

ICU = Intensive Care Unit, CS = Cardiac Surgery, CCU = Coronary Care Unit and TR = Trauma.∩ denotes a group of patients that include subjects belonging to two or more high-risk groups.

Table 2 details the scenarios simulated in this study; a brief description of the scenario is given in second column; third column shows the percentages of RBC units transfused to each of the HRGs and BBRs and the last columns defines the allocation method used for each group. For example, in Scenario 2, any ICU patient is determined by adding the percentage of any mutually exclusive group from Table 1 that includes ICU patients. We applied the TM to model the effect on US blood supply of transfusing the HRGs with the corresponding ‘threshold’ age of RBCs we assumed from the literature [1–6] (i.e., age of RBCs ≤7 days for ICU, ≤14 days for CS and CCU patients and ≤28 days for TR patients). For scenarios in which two or more HRGs were combined, the TM attempted to allocate the younger age of RBCs to the overlapping group, i.e., in Scenario 6, Table 2 we applied TM7 to ICU and TM14 to CS, while we assigned TM7 to ICU ∪ CS.

**Table 2:** Percentage of total RBC units transfused and reported by the CMS database for the period 2007-2012 by type of scenario. Scenario illustration (Venn diagrams), group description, and allocation method used for each scenario are also presented.

Type of scenario	Group Description	Percentage of total RBC units transfused during 2007-2012	Allocation Method ^a^
**1. Baseline (BBRs-LO)**	All blood recipients receive current standard of care.	100%	LO
**2. ICU**	Any ICU patients	43.2%	TM7
	BBRs	56.8%	LO
**3. CS**	Any CS patients	33.2%	TM14
	BBRs	66.8%	LO
**4. CCU**	Any CCU patients	14.6%	TM14
	BBRs	85.4%	LO
**5. TR**	Any Trauma patients	5.2%	TM28
	BBRs	94.8%	LO
**6. ICU ∪ CS** ^**b**^ ^**c**^ ^**d**^	Any ICU patients + non-overlapping CS patients.	ICU = 43.2%, CS = 11.7%	TM7
		ICU ∪ CS = 54.9%	TM14
	BBRs	45.1%	LO
**8. All HRGs- TM7All HRGs- TM14**	All HRGs combined	62%	TM7 or TM14
	BBRs	38%	LO

ICU = Intensive Care Unit, CS = Cardiac Surgery, CCU = Coronary Care Unit, TR = Trauma and BBRs = Baseline Blood Recipients. a LO = Likely Oldest allocation method (1). TM7, TM14 and TM28 = ‘Threshold Method’, with age of RBCs equal or less than 7, 14, and 28 days, respectively. The ‘threshold’ ages of RBCs were recommended from literature[3,6].

b ∪ denotes union of two or more HRGs.

c ∩ denotes intersection between two HRGs.

d $\bar{ICU}$, denotes the complement of this group.

## Results

To evaluate the impact on supply of transfusing a subset of HRGs with fresher stored RBCs, we compared the baseline blood recipients to several selected scenarios (Scenario1 vs. 2-8, Table 2). In Scenario 1 we assumed all patients would receive older RBC units using the LO allocation method [10], while in Scenarios 2 to 8 high-risk patients were given RBCs of a certain younger age (TM). In Table 2, we presented only a subset of the scenarios we simulated (for more details, see additional simulated scenarios, S6 Table and S4–S7 Figs). We set the same initial conditions, i.e., the collector bank, the hospital bank, the daily-predicted donations and transfusions, for each independent simulated scenarios. For each scenario, we estimated the total AAD number of RBC units available in the US blood system (Collector + Hospital) either for all blood types or by blood type.

**Fig 1:**
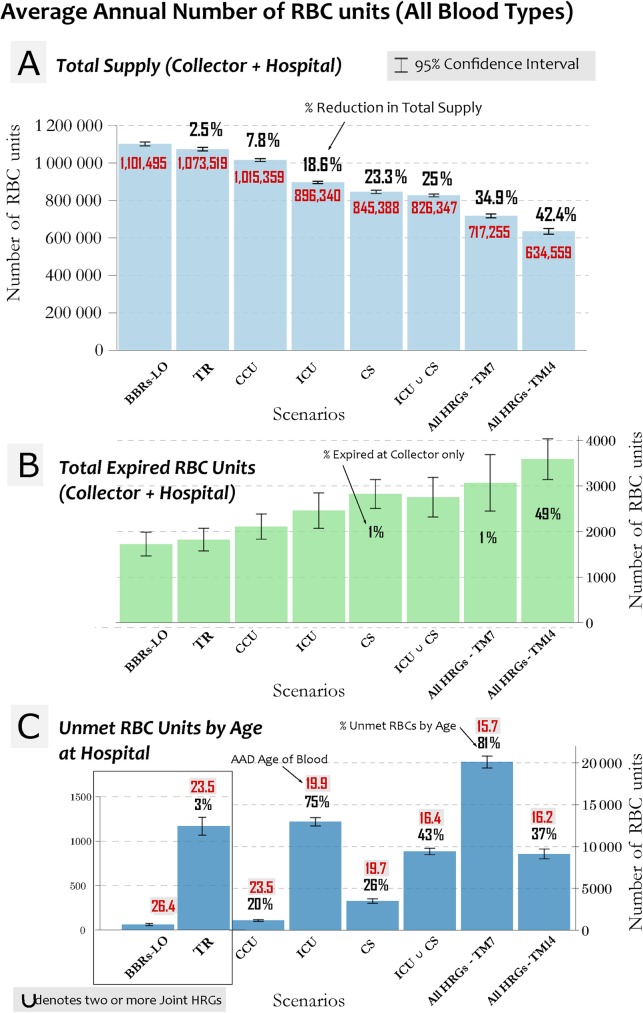
**The Annual Average Daily (AAD) numberof RBC units available in the system for: (A) Total Supply (collector + hospital, light blue bars), AAD estimated number of RBC units (red), and percent reduction in total supply with respect to baseline scenario (BBRs-LO) (above bars forscenarios 2 to 8); (B) Expired RBC Units, (collector + hospital, green bars) and AAD number of expired units at collectoronly are shown on bars; (C) AAD Unmet RBC Units by Age at the hospital (dark blue bars), the percentage of unmet units by age (above bars), and AAD age of the transfused blood at the hospital (above bars, red on grey background).** The scenarios are sorted in a descending order according to their total supply. Error bars represent 95% confidence intervals. AAD = annual average daily.

Fig 1(A) illustrates the changes in the percent reduction of the AAD number of RBC units available for each scenario with respect to baseline scenario (BBRs-LO). We sorted scenarios in descending order according to the total blood supply levels. We observed 2.5% and 7.8% reduction in total supply levels for TR (representing 5.2% of total RBC units transfused, TM28) and CCU patients (14.6% of total RBC transfused, TM14), respectively. We observed a reduction in supply of 18.6% and 23.3% for ICU (43.2%, TM7) and CS patients (33.2%, TM14), respectively. The most extreme scenarios, All-HRGs TM7 and TM14, reduced the total available supply levels by up to 42% compared to baseline.

Fig 1(B) shows the percent reduction in the AAD number of expired RBC units corresponding to each scenario of Fig 1(A). We observed the fewest expired RBC units in BBRs-LO scenario (AAD of 1,725 units, 95% CI: 1,466–1,985); while the highest expiration occurred in All HRGs-TM14 scenario (AAD of 3,585 units, 95% CI: 3,191-3,979) showing a 52% increase in the number of expired units compared to baseline.

Fig 1(C) shows for each scenario the AAD number of unmet RBC units by age at the hospital level, the percentage of unmet RBC units by age; and the AAD age of transfused RBCs at the hospital level. We observed a wide range of unmet RBC units by age from a daily average of 60 unmet units (3%) for TR scenario, to approximately a daily average of 20,000 unmet units (81%) for the All HRGs-TM7 scenario. Fig 1(C) shows also the AAD age of the overall transfused blood (BBRs+HRGs) at the hospital, which measured the effect of the allocation method on the AAD age among the different scenarios. The AAD age of the overall transfused RBCs ranged from 26.4 days for the baseline (BBRs-LO) scenario to 15.7 days for the All-HRGs TM7 scenario.

**Fig 2:**
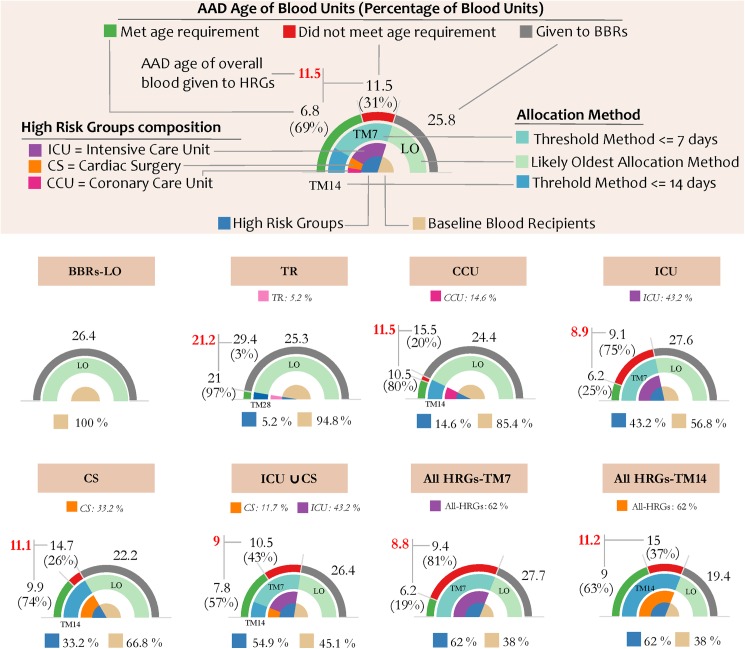
The annual average daily (AAD) age of transfused blood units for each simulated scenario listed in Table 2. We provide a description of each element of the half-pie chart from the inner to the outer layer, as follows: 1) percentage of total RBC units transfused among elderly for the overall HRGs (blue) versus BBRs (tan); 2) percentage of total RBC units transfused partitioned by HRGs (purple, orange and pink); 3) allocation method used foreach recipient group (HRGs – different shades of light blue; BBRs in teal); 4) AAD age of the blood transfused to each recipient group and the percentage of ‘met’ (green) and ‘unmet’ (red) RBC units by age forHRGs only (in parenthesis); and 5) AAD age of the overall transfused blood given to HRGs (bold red).

In Fig 2, we designed a half-pie chart for each scenario reported in Table 2 (Scenarios 1-8). In those scenarios in which we applied TM7 only to the HRGs, the AAD age of RBCs transfused to the BBRs was higher—ICU (27.6 days) and All HRGs-TM7 (27.7 days)—compared to BBRs-LO (26.4 days). However, when we applied TM14 only to the HRGs, the AAD age of RBCs for BBRs reduced, as observed in CCU scenario (24.4 days), CS scenario (22.2 days) and All HRGs-TM14 (19.4 days). In addition, we observed in Fig 2 that the actual AAD age of the overall transfused RBCs allowed by the system was about 8.8 days when we applied TM7, i.e., ICU and All HRGs-TM7 scenarios. However, the actual AAD age of the overall transfused RBCs allowed by the system when we applied TM14 ranged from 11.1 to 11.5, i.e., CS and CCU scenarios. Results on the performance of the blood system in meeting the desired ‘threshold’ age of transfused RBCs suggest an overall higher performance of TM14 compared to TM7, although the system likely expires more RBC units. For each scenario, in order to assess the efficiency of the system, we explored the percentage of met RBC units by age (i.e., 100%—percentage of unmet RBC units by age) to the HRGs, and the average age of RBC transfused to the HRG (numbers shown in bold red, Fig 2). If the percentage of met RBC units by age was greater than 50%, then the age of the transfused RBC units would be below the considered age threshold. This holds true only for those scenarios where a single age threshold was applied. For example, the scenarios that implemented TM7 only, i.e., ICU and All HRGs TM7, we found that the efficiency of the system was only 25% and 19%, respectively. In both scenarios, the system failed to provide the RBC units stored for 7 days or less, in fact, the average age of transfused RBC units was 8.9 days for ICU and 8.8 days for All HRGs TM7. On the other side, those scenarios that implemented TM14 only, i.e., CS and All HRGs-TM14, the efficiency of the system was 74% and 63%, respectively, with the average age of the RBC units 11.1 days and 11.2 days. In the case where two or more TMs were implemented, the average age of transfused RBC units to the HRGs is less clear. The percentage of met units by age could still be used to assess the system efficiency without giving details about a specific HRG.

## Discussion

We developed the ‘Threshold Method’ to investigate the impact on the US blood supply levels of preferentially transfusing fresher stored RBCs to the higher risk critically ill patients. The selected scenarios represented plausible examples of the high-risk blood recipients who might benefit from receiving fresher stored RBC units. We expected to see a proportional relationship between the age of RBCs required for transfusion and the total blood supply levels available in the system, i.e., the younger the age of RBCs required, the lower the supply levels. However, we observed a counterintuitive finding when we compared the two scenarios All HRGs-TM7 and All HRGs-TM14 both representing 62% of total RBC units transfused. The estimated AAD total supply for scenario All HRGs-TM14 was 11.5% lower than that of All HRGs-TM7. By comparing Fig 1(A) to Fig 1(B), we observed a nearly inversely proportional relationship between the total supply level for each scenario and the corresponding number of expired units. Because each scenario satisfied the same initial conditions for the daily supply and demand, the reduction we observed in the total supply of scenario All HRGs-TM14 could not be attributed to a higher demand, instead, to a higher number of expired units (Fig 1(B)). For the non steady-state simulation presented in this paper, we selected the average final banks from among 100 steady-state simulations to be the initial banks for the collector and hospital. Using the same initial banks, for each scenario presented, we ran 500 independent one-year (365 days) simulations.

These findings are in contrast with previous results reported by Simonetti et al. 2014 [10] where no statistically significant difference was found in the number of expired RBCs among the different allocation methods (i.e., FIFO, LO and LN). Moreover, if changes in the transfusion practices take place, a higher demand for fresher stored RBC units will follow to accommodate the HRGs needs. However, in our simulation we observed that the system, due to the scarce availability of RBC units with required age ≤7 days when implementing TM7, tended to allocate more frequently RBC units that were older than the required age. Conversely, in the relatively relaxed situation of TM14 (required age ≤14 days), the system was able to supply the demand for fresher stored RBC units for a longer time; while leaving older RBC units to expire more. The new TM allocation method in preferentially transferring fresher stored RBC units from the collector to the hospital and then to the HRG recipient in a not-FIFO way, no longer satisfy previous modeling [10] conditions of a traditional queuing system. The current model is a non-adaptive model where we stochastically sampled daily supply and demand from historic data to illustrate any potential deviation the system would face from the status-quo. In future work, we may consider implementing an adaptive framework to respond to changes in the inventory, and thus mimic practical inventory management techniques.

Model findings suggest that the behavior of the blood supply system may depend on both the amount of blood needed for transfusion to a HRG, i.e., the percentage of blood utilized to the HRG, and on the blood requirement, i.e., the age of RBC units. Both factors play an important role in predicting the transient impact on the blood supply levels during the first year following any potential changes in transfusion practice.

However, the estimated number of units available and those expired cannot provide a sufficient evaluation on the impact on the blood supply if fresher stored RBC units are preferentially transfused to a specific subset of critically ill patients. In order to provide a more accurate measure of the performance of the US blood supply system, it is important to understand the capability of the system in delivering the required RBC units with respect to the specified age, or ‘unmet RBC units by age.’ We found no clear trend between the total supply (Fig 1(B)) and the number of unmet RBC units by age (Fig 1(C)). However, we observed a negative correlation between the ratio of TM7 to TM14 and the percentage of unmet RBC units by age. For example, when comparing scenarios ICU with ICU ⋃ CS (Table 2 and Fig 1(C)), we estimated 43% of unmet RBC units by age for ICU ⋃ CS compared to the 75% estimated in ICU, despite the higher total amount of units transfused to the HRGs (54.9%). In general, despite a larger amount of units transfused to HRGs, we estimated less unmet RBC units by age, when the proportion of the HRGs assigned to TM14 were larger compared to that assigned to TM7, i.e., the performance of the system declines when fresher stored RBCs is constantly required.

All the scenarios in our study were based on data extracted from the CMS database, which is limited to the blood utilization among the US elderly inpatients. The elderly population, in addition to utilizing ~58% of the total blood utilized in the US [24], is more vulnerable to infections, hospitalizations, higher prevalence of chronic diseases [25]. Therefore, ascertainment of blood utilization for HRGs, especially for trauma patients [26], in the general US population might be useful to more accurately model the HRGs blood recipients and further predict their impact on the blood supply.

To conclude, we learned from this study how difficult it may be to identify an ideal scenario where the age-specific demand of RBCs is met, while the reduction in the US blood supply is acceptable. Only considering the three measures of performance (i.e., total supply, expired RBC units and unmet RBC units by age) in context, we may help understanding the effect on the US blood supply if transfusing fresher storedRBC units is proven beneficial to improve recipient outcomes. However, under current assumptions, we demonstrated how the newly developed allocation method allows a relatively efficient distribution of blood while preferentially providing fresher stored RBCs to the high-risk blood recipient except for some disruptions in the US blood supply observed in certain simulated scenarios. Most importantly, implementing ‘ad-hoc’ models can help assist stakeholders and the blood community to make informed decisions that may impact the availability of blood in the US blood supply.

## Supporting information

S1 Fig **A flowchart of the ‘Threshold Method’ (TM) algorithm to illustrate the mechanism of allocating blood into the system 1) from the Hospital to the HRGs recipients; and 2) from the Collector Bank to the Hospital, on each day of the Monte Carlo simulation**.
(DOCX)

S2 Fig Multinomial-Dirichlet distribution with shape parameters *α*_*i*_ × 1,000. (DOCX)

S3 Fig Multinomial-Dirichlet distribution with shape parameters *α*_*i*_ × 10,000. (DOCX)

S4 Fig **The Annual Average Daily (AAD) number of RBC units available in the system for: (A) Total Supply (collector+ hospital, tan bars), average values (red), and percent reduction in total supply with respect to baseline scenario (BBR-LO) (above bars for scenarios 2 to 11); (B) Expired RBC Units, (collector +hospital, green bars) and percentage of expired units at collector only are shown on bars; (C) Unmet RBC Units by Age by blood type at the hospital, the percentage of unmet units by age (above bars), and mean age of the transfused blood at the hospital (above bars, red on grey background).** The scenarios are sorted in a descending order according to their total supply. Error bars represent 95% confidence intervals.”
(DOCX)

S5 Fig Mean age of transfused blood units for each simulated scenario listed in Table 2. Description of each element of the half-pie chart from inner to the outer layer as follows: 1) percentage of total RBC units transfused among elderly for the overall HRGs (blue) versus BBR (tan); 2) percentage of total RBC units transfused decomposed by HRGs (purple, orange and pink); 3) allocation method used for each recipient group (HRGs – different shades of light blue; BBR in teal); 4) mean age of the blood transfused to each recipient group and the percentage of ‘met’ (green) and ‘unmet’ (red) RBC units by age for HRGs only (in parenthesis); and 5) mean age of the overall transfused blood given to HRGs (bold red).
(DOCX)

S6 Fig The Annual Average Daily number of RBC units available in the system (or, total supply, collector+ hospital, tan bars) for the additional scenarios BBR-TM7, BBR-TM14 using “Threshold Method” (left), and Shelf-Life (SL) scenarios SL-35, SL-28, SL-28, SL-21, SL-14 and SL-7 using ”Likely Oldest” (right). The mean age of blood for the overall transfused blood at the hospital is shown above the bars.
(DOCX)

S7 Fig The Annual Average Daily number of expired RBC units, (collector+ hospital, tan bars) for the additional scenarios BBR-TM7 and BBR-TM14 using “Threshold Method” (left), and Shelf-Life (SL) scenarios SL-35, SL-28, SL-28, SL-21, SL-14 and SL-7 using ”Likely Oldest” (right). (DOCX)

S1 Table Standard Phenotype-compatibility rules used in blood cross-matching in between Collector and Hospitals. (DOCX)

S2 Table Practical Phenotype-compatibility rules used in blood cross-matching in Hospitals to transfuse Patients. (DOCX)

S3 Table Annual average daily number of RBC units for all blood types combined and by ABO/Rh. (DOCX)

S4 Table ICD-9-CM DX codes and their description used to identify Trauma patients from the CMS data. (DOCX)

S5 Table ICD-9-CM Procedure codes used to identify cardiac patients from the CMS data. (DOCX)

S6 Table Percentage of total RBC units transfused and reported by the CMS database for the period 2007-2012 by type of scenario. Scenario illustration, group description, and allocation method used for each scenario are also presented.
(DOCX)
