# Correction: Modeling the potential impact on the US blood supply of transfusing critically ill patients with fresher stored red blood cells

**DOI:** 10.1371/journal.pone.0176731

**Published:** 2017-04-24

**Authors:** The PLOS ONE Staff

Hussein Ezzeldin (HE) is incorrectly listed as one of the persons providing supervision in the Author Contributions section. Arianna Simonetti (AS) should be listed as the person who provided supervision. The publisher apologizes for the error.

In Table 2, the final row of the column “Type of Scenario” is incorrect. There is also an error in the caption for Table 2. Please see the corrected Table 2 and caption here.

**Table 2:** Percentage of total RBC units transfused and reported by the CMS database for the period 2007–2012 by type of scenario. Scenario illustration (Venn diagrams), Type of scenario, group description, and allocation method used for each scenario are also presented.

Type of scenario	Group Description	Percentage of total RBC units transfused during 2007–2012	Allocation Method ^a^
**1. Baseline (BBRs-LO)**	All blood recipients receive current standard of care.	100%	LO
**2. ICU**	Any ICU patients	43.2%	TM7
	BBRs	56.8%	LO
**3. CS**	Any CS patients	33.2%	TM14
	BBRs	66.8%	LO
**4. CCU**	Any CCU patients	14.6%	TM14
	BBRs	85.4%	LO
**5. TR**	Any Trauma patients	5.2%	TM28
	BBRs	94.8%	LO
**6. ICU ∪ CS** ^b^ ^c^ ^d^	Any ICU patients + non-overlapping CS patients.	ICU = 43.2%, CS = 11.7%	TM7
		ICU ∪ CS = 54.9%	TM14
	BBRs	45.1%	LO
**7. All HRGs- TM7** **or** **8. All HRGs- TM14**	All HRGs combined	62%	TM7 or TM14
	BBRs	38%	LO

ICU = Intensive Care Unit, CS = Cardiac Surgery, CCU = Coronary Care Unit, TR = Trauma and BBRs = Baseline Blood Recipients.

^a^ LO = Likely Oldest allocation method (1). TM7, TM14 and TM28 = ‘Threshold Method’, with age of RBCs equal or less than 7, 14, and 28 days, respectively. The ‘threshold’ ages of RBCs were recommended from literature [3,6].

^b^ ∪ denotes union of two or more HRGs.

^c^ ∩ denotes intersection between two HRGs.

^d^ $\bar{\text{ICU}}$, denotes the complement of this group.
