# Supplementary material for: Modeling the potential impact on the US blood supply of transfusing critically ill patients with fresher stored red blood cells
Source: PLoS One. 2017 Mar 20;12(3):e0174033. doi: 10.1371/journal.pone.0174033 (PMC5358863; doi:10.1371/journal.pone.0174033)
Supplement: S1 Fig — A flowchart of the ‘Threshold Method’ (TM) algorithm to illustrate the mechanism of allocating blood into the system 1) from the Hospital to the HRGs recipients; and 2) from the Collector Bank to the Hospital, on each day of the Monte Carlo simulation. (DOCX) [file pone.0174033.s001.docx]

**Supporting Material**

**S1 Fig. A flowchart of the ‘Threshold Method’ (TM) algorithm to illustrate the mechanism of allocating blood into the system 1) from the Hospital to the HRGs recipients; and 2) from the Collector Bank to the Hospital, on each day of the Monte Carlo simulation.**

Average daily demand of RBCs for each HRG and required threshold age disaggregated by blood phenotype

For each blood type

**Yes**

Transfer blood to HRG patients

**No**

**On each day**

Check exact blood match availability by age

Check compatible blood availability by age

**Yes**

**Yes**

**No**

Check the next available younger exact or compatible blood type

Recorded as *‘unmet blood units by age’*

**No**

Recorded as *‘shortage in blood units’*

For each blood type

**Yes**

**No**

Check available exact or compatible blood type

Transfer blood to BBR patients

BBR = Baseline Blood Recipients, HRG = High Risk Group, LO = Likely Oldest, RBCs = Red Blood Cells, TM = Threshold Method

Average daily demand of RBCs for each HRG and required threshold age disaggregated by blood phenotype for next day

For each blood type

**Yes**

Transfer blood to Hospital

**No**

Submit request to Collector Bank to fulfill the deficit

**Yes**

**Yes**

**No**

**Hospital checks for 6 days reserve**

**Yes**

Transfer blood to Hospital bank

Submit request to Collector Bank to maintain 6 days

Collector bank checks for compatible blood availability

**Yes**

End of day

**No**

Hospital bank checks for exact blood type match availability by age

Collector Bank checks for compatible blood type availability by age
