# Supplementary figures and images for: Modeling the potential impact on the US blood supply of transfusing critically ill patients with fresher stored red blood cells
Source: PLoS One. 2017 Mar 20;12(3):e0174033. doi: 10.1371/journal.pone.0174033 (PMC5358863; doi:10.1371/journal.pone.0174033)

| 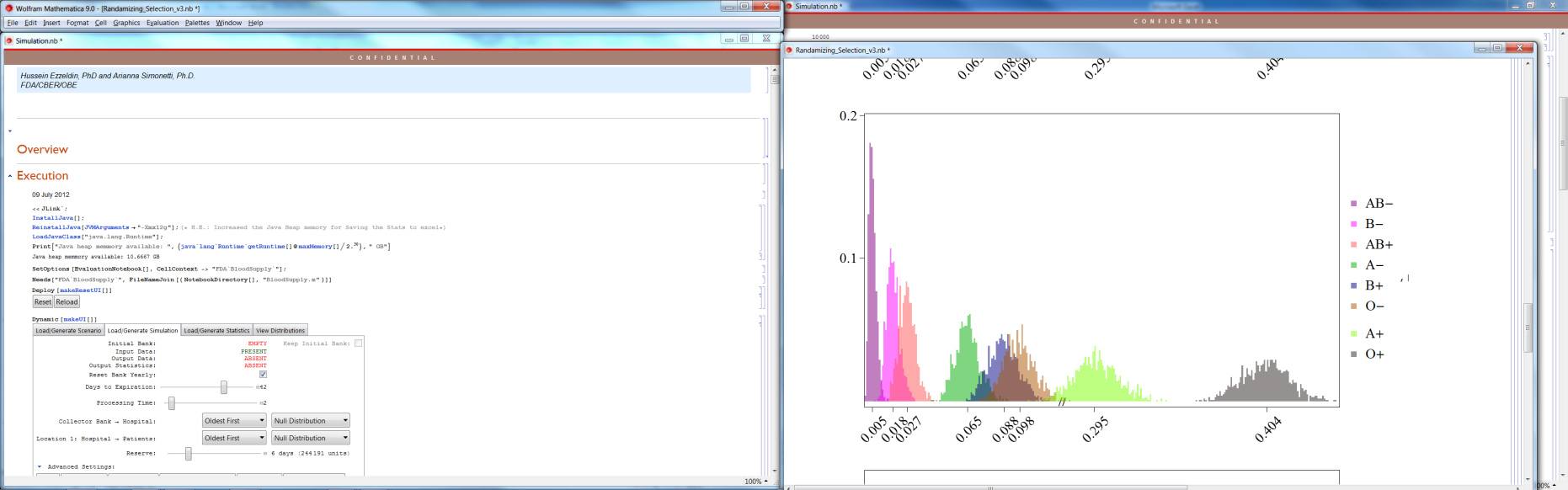  **S2 Fig. Multinomial-Dirichlet distribution with shape parameters** |  |
| --- | --- |

Supplement: S2 Fig — (DOCX) [file pone.0174033.s002.docx]

| 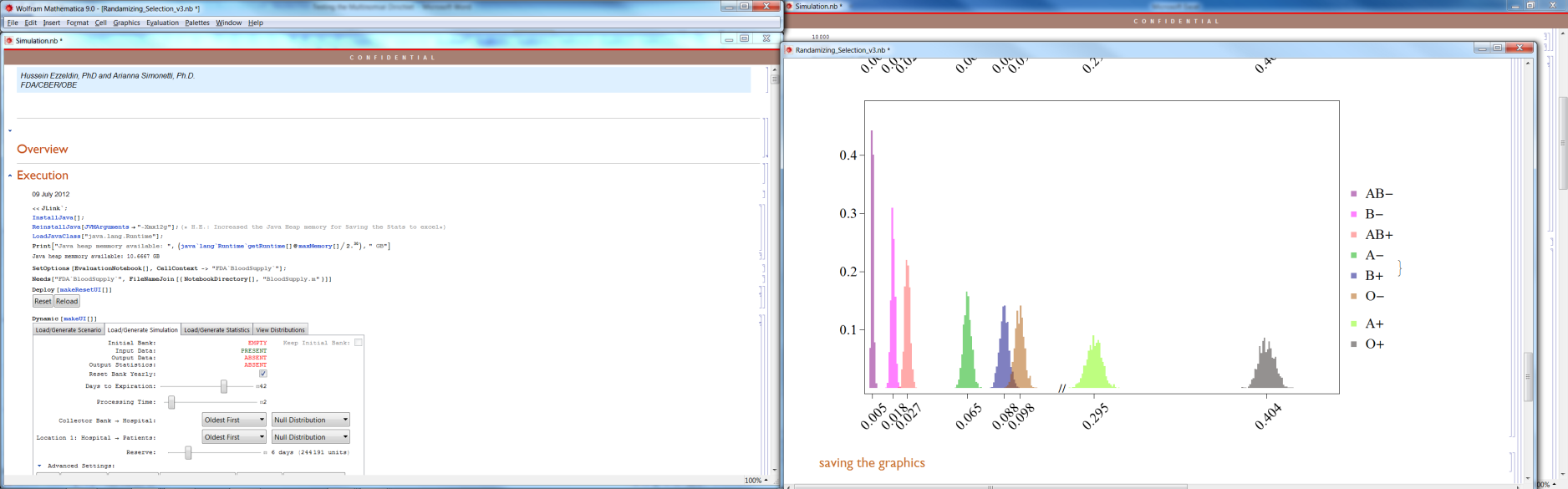  **S3 Fig. Multinomial-Dirichlet distribution with shape parameters** |  |
| --- | --- |

Supplement: S3 Fig — (DOCX) [file pone.0174033.s003.docx]
