# Supplementary material for: Modeling the potential impact on the US blood supply of transfusing critically ill patients with fresher stored red blood cells
Source: PLoS One. 2017 Mar 20;12(3):e0174033. doi: 10.1371/journal.pone.0174033 (PMC5358863; doi:10.1371/journal.pone.0174033)
Supplement: S4 Fig — The Annual Average Daily (AAD) number of RBC units available in the system for: (A) Total Supply (collector+ hospital, tan bars), average values (red), and percent reduction in total supply with respect to baseline scenario (BBR-LO) (above bars for scenarios 2 to 11); (B) Expired RBC Units, (collector +hospital, green bars) and percentage of expired units at collector only are shown on bars; (C) Unmet RBC Units by Age by blood type at the hospital, the percentage of unmet units by age (above bars), and mean age of the transfused blood at the hospital (above bars, red on grey background). The scenarios are sorted in a descending order according to their total supply. Error bars represent 95% confidence intervals.” (DOCX) [file pone.0174033.s004.docx]

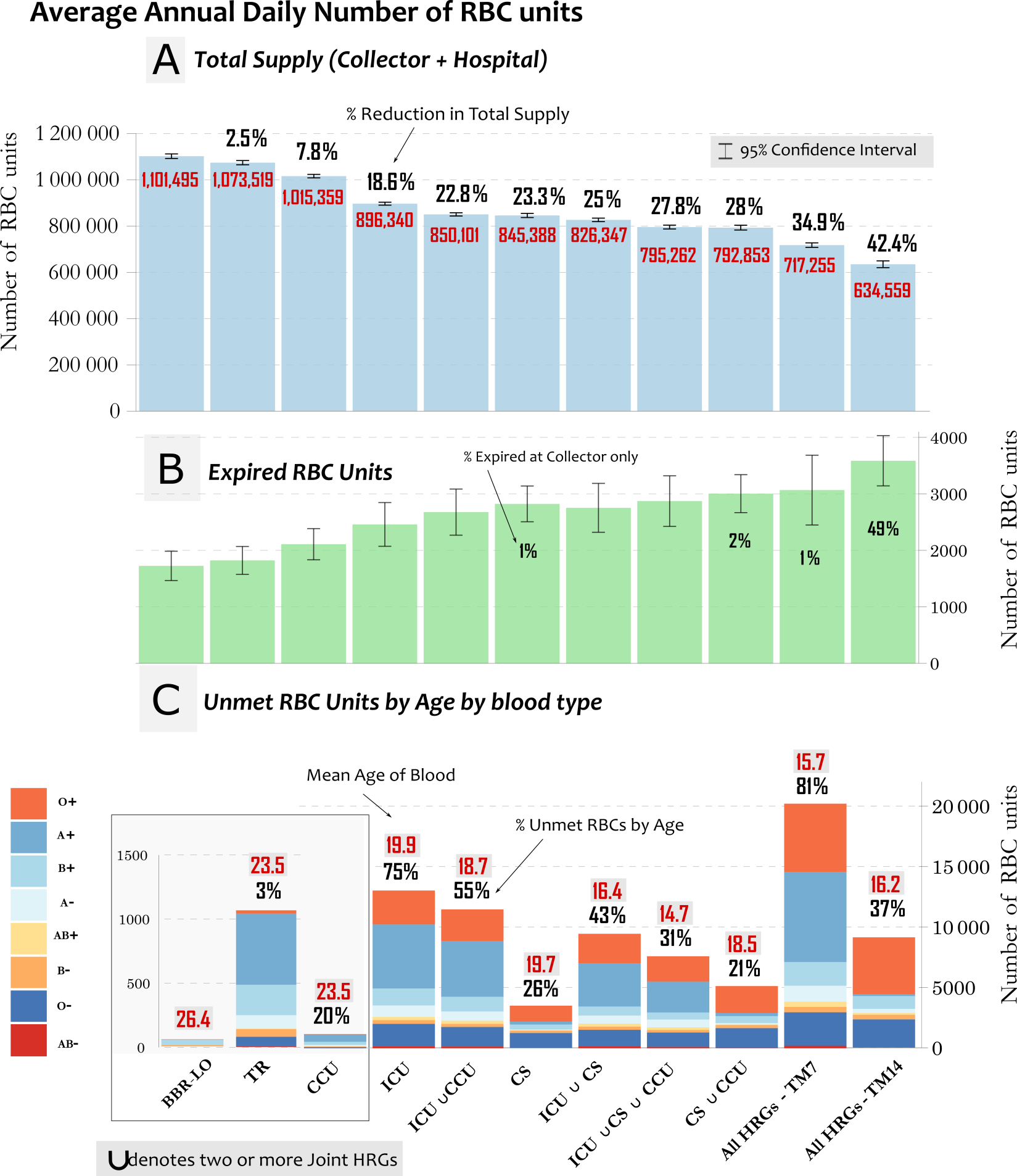


**S4 Fig. The Annual Average Daily (AAD) number of RBC units available in the system for: (A) Total Supply (collector + hospital, light blue bars), mean values (red), and percent reduction in total supply with respect to baseline scenario (BBR-LO) (above bars for scenarios 2 to 11); (B) Expired RBC Units, (collector + hospital, green bars) and percentage of expired units at collector only are shown on bars; (C) Unmet RBC Units by Age by blood type at the hospital, the percentage of unmet units by age (above bars), and mean age of the transfused blood at the hospital (above bars, red on grey background).** The scenarios are sorted in a descending order according to their total supply. Error bars represent 95% confidence intervals.
