# Supplementary material for: Modeling the potential impact on the US blood supply of transfusing critically ill patients with fresher stored red blood cells
Source: PLoS One. 2017 Mar 20;12(3):e0174033. doi: 10.1371/journal.pone.0174033 (PMC5358863; doi:10.1371/journal.pone.0174033)
Supplement: S5 Fig — Description of each element of the half-pie chart from inner to the outer layer as follows: 1) percentage of total RBC units transfused among elderly for the overall HRGs (blue) versus BBR (tan); 2) percentage of total RBC units transfused decomposed by HRGs (purple, orange and pink); 3) allocation method used for each recipient group (HRGs – different shades of light blue; BBR in teal); 4) mean age of the blood transfused to each recipient group and the percentage of ‘met’ (green) and ‘unmet’ (red) RBC units by age for HRGs only (in parenthesis); and 5) mean age of the overall transfused blood given to HRGs (bold red). (DOCX) [file pone.0174033.s005.docx]

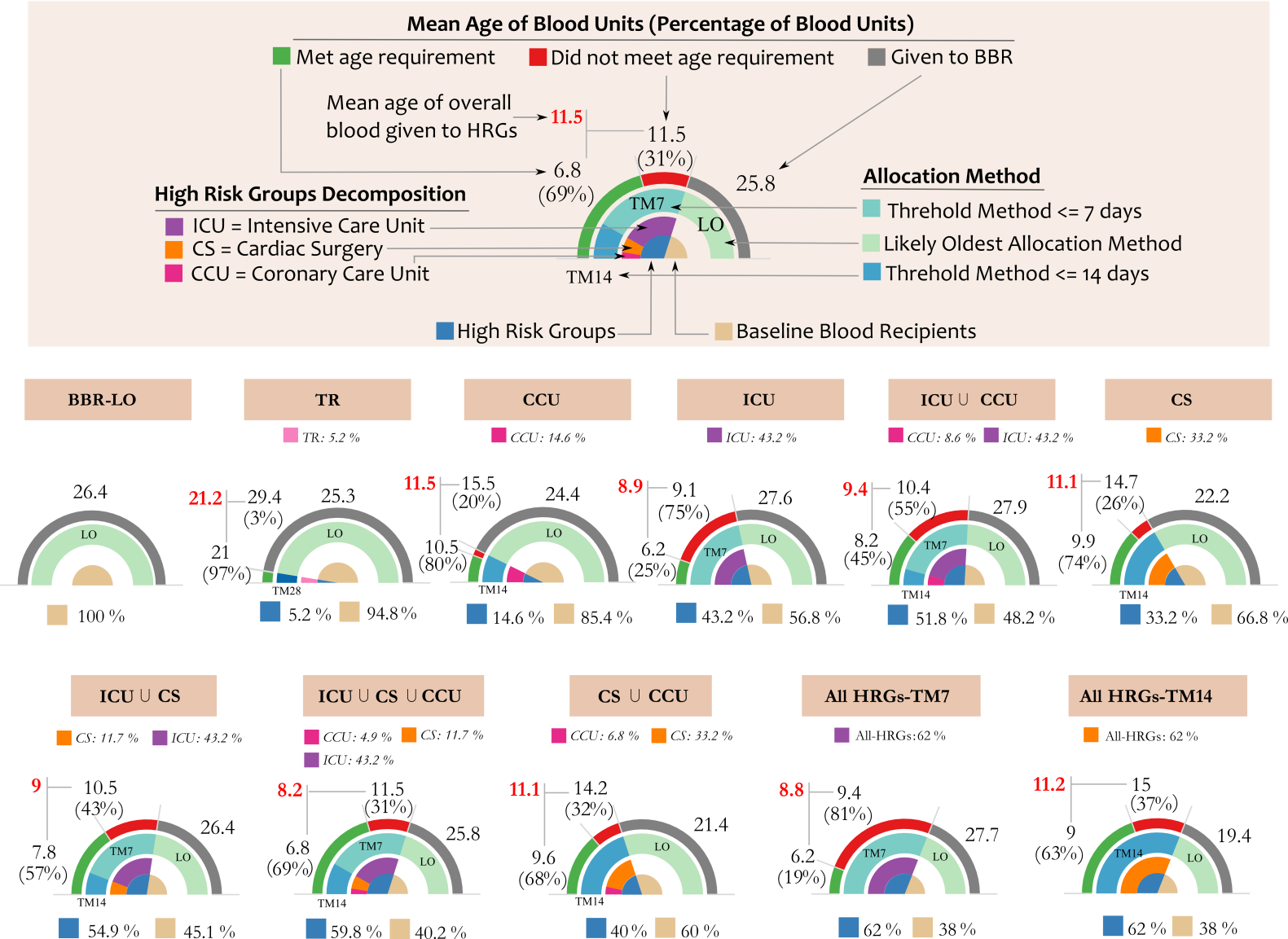


S5 Fig. Mean age of transfused blood units for each simulated scenario listed in Table 2. Description of each element of the half-pie chart from inner to the outer layer as follows: 1) percentage of total RBC units transfused among elderly for the overall HRGs (blue) versus BBR (tan); 2) percentage of total RBC units transfused decomposed by HRGs (purple, orange and pink); 3) allocation method used for each recipient group (HRGs – different shades of light blue; BBR in teal); 4) mean age of the blood transfused to each recipient group and the percentage of ‘met’ (green) and ‘unmet’ (red) RBC units by age for HRGs only (in parenthesis); and 5) mean age of the overall transfused blood given to HRGs (bold red).
