# Supplementary material for: Modeling the potential impact on the US blood supply of transfusing critically ill patients with fresher stored red blood cells
Source: PLoS One. 2017 Mar 20;12(3):e0174033. doi: 10.1371/journal.pone.0174033 (PMC5358863; doi:10.1371/journal.pone.0174033)
Supplement: S6 Fig — The mean age of blood for the overall transfused blood at the hospital is shown above the bars. (DOCX) [file pone.0174033.s006.docx]

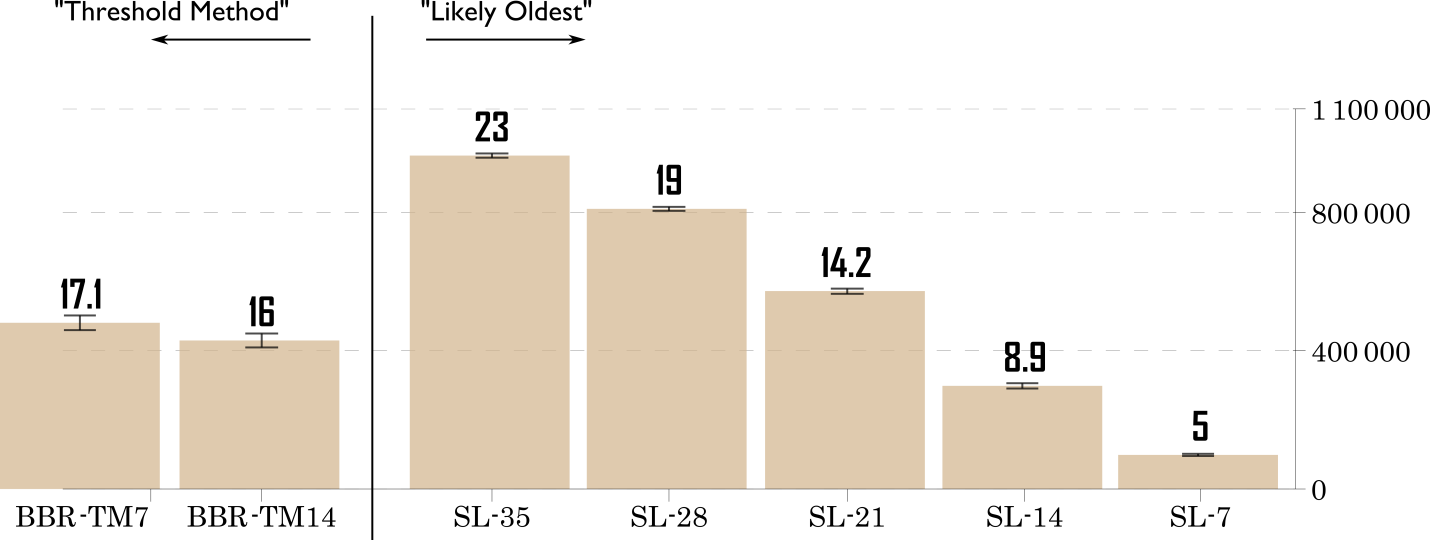


**S6 Fig. The Annual Average Daily number of RBC units available in the system (or, total supply, collector+ hospital, tan bars) for the additional scenarios BBR-TM7, BBR-TM14 using “Threshold Method” (left), and Shelf-Life (SL) scenarios SL-35, SL-28, SL-28, SL-21, SL-14 and SL-7 using ”Likely Oldest” (right). The mean age of blood for the overall transfused blood at the hospital is shown above the bars.**
