# Supplementary material for: Modeling the potential impact on the US blood supply of transfusing critically ill patients with fresher stored red blood cells
Source: PLoS One. 2017 Mar 20;12(3):e0174033. doi: 10.1371/journal.pone.0174033 (PMC5358863; doi:10.1371/journal.pone.0174033)
Supplement: S1 Table — (DOCX) [file pone.0174033.s008.docx]

S1 Table. Standard Phenotype-compatibility rules used in blood cross-matching in between Collector and Hospitals.

| Blood type | Phenotype compatibility table optimized by phenotype order of preference | | | | | | | |
| --- | --- | --- | --- | --- | --- | --- | --- | --- |
| O+ | O+ | O- |  |  |  |  |  |  |
| O- | O- |  |  |  |  |  |  |  |
| A+ | A+ | A- | O+ | O- |  |  |  |  |
| A- | A- | O- |  |  |  |  |  |  |
| B+ | B+ | O+ | B- | O- |  |  |  |  |
| B- | B- | O- |  |  |  |  |  |  |
| AB+ | AB+ | AB- | A+ | A- | B+ | B- | O+ | O- |
| AB- | AB- | A- | B- | O- |  |  |  |  |
