# Supplementary material for: Modeling the potential impact on the US blood supply of transfusing critically ill patients with fresher stored red blood cells
Source: PLoS One. 2017 Mar 20;12(3):e0174033. doi: 10.1371/journal.pone.0174033 (PMC5358863; doi:10.1371/journal.pone.0174033)
Supplement: S2 Table — (DOCX) [file pone.0174033.s009.docx]

S2 Table. Practical Phenotype-compatibility rules used in blood cross-matching in Hospitals to transfuse Patients.

| Blood type | Phenotype compatibility table optimized by phenotype order of preference | | | | | | | |
| --- | --- | --- | --- | --- | --- | --- | --- | --- |
| O+ | O+ | O- |  |  |  |  |  |  |
| O- | O- | O+ |  |  |  |  |  |  |
| A+ | A+ | O+ | A- | O- |  |  |  |  |
| A- | A- | O- | A+ | O+ |  |  |  |  |
| B+ | B+ | O+ | B- | O- |  |  |  |  |
| B- | B- | O- | B+ | O+ |  |  |  |  |
| AB+ | AB+ | A+ | B+ | O+ | AB- | A- | B- | O- |
| AB- | AB- | A- | B- | O- | AB+ | A+ | B+ | O+ |
