# Supplementary material for: Modeling the potential impact on the US blood supply of transfusing critically ill patients with fresher stored red blood cells
Source: PLoS One. 2017 Mar 20;12(3):e0174033. doi: 10.1371/journal.pone.0174033 (PMC5358863; doi:10.1371/journal.pone.0174033)
Supplement: S3 Table — (DOCX) [file pone.0174033.s010.docx]

S3 Table. Annual average daily number of RBC units for all blood types combined and by ABO/Rh.

| Scenario  Parameter | | Baseline | | | Scenario 1  α_prev_ ×1,000 | | | Scenario 2  α_prev_ ×10,000 | | |
| --- | --- | --- | --- | --- | --- | --- | --- | --- | --- | --- |
|  |  | Mean | 95% CI | | Mean | 95% CI | | Mean | 95% CI | |
| All blood types  cm | | 989,907 | 979,077 | 1,000,737 | 995,360 | 984,637 | 1,006,083 | 1,001,736 | 990,842 | 1,012,629 |
| By ABO/Rh | O+ | 516,525 | 510,499 | 522,551 | 519,970 | 513,498 | 526,442 | 527,945 | 521,589 | 534,301 |
|  | O- | 18,878 | 17,397 | 20,358 | 22,201 | 20,420 | 23,982 | 17,256 | 16,006 | 18,506 |
|  | A+ | 156,394 | 151,834 | 160,953 | 158,060 | 152,764 | 163,356 | 156,361 | 151,595 | 161,127 |
|  | A- | 103,419 | 102,387 | 104,451 | 103,083 | 102,064 | 104,101 | 102,431 | 101,460 | 103,402 |
|  | B+ | 138,386 | 137,006 | 139,766 | 138,434 | 137,040 | 139,828 | 136,970 | 135,641 | 138,299 |
|  | B- | 22,414 | 22,169 | 22,659 | 24,842 | 24,501 | 25,183 | 21,367 | 21,107 | 21,627 |
|  | AB+ | 33,589 | 33,221 | 33,956 | 28,432 | 28,015 | 28,849 | 39,092 | 38,711 | 39,473 |
|  | AB- | 303 | 282 | 324 | 339 | 311 | 368 | 314 | 290 | 337 |
