# Supplementary material for: Modeling the potential impact on the US blood supply of transfusing critically ill patients with fresher stored red blood cells
Source: PLoS One. 2017 Mar 20;12(3):e0174033. doi: 10.1371/journal.pone.0174033 (PMC5358863; doi:10.1371/journal.pone.0174033)
Supplement: S4 Table — (DOCX) [file pone.0174033.s011.docx]

**S4 Table. ICD-9-CM DX codes and their description used to identify Trauma patients from the CMS data.**

| **ICD-9-CM**  **DX codes** | **Diagnosis description** |
| --- | --- |
| 800-804 | Fracture Of Skull, face bones or one of the two with other bones |
| 805-809 | Fracture Of Spine And Trunk |
| 850-854 | Intracranial Injury, Excluding Those With Skull Fracture |
| 860-869 | Internal Injury Of Chest, Abdomen, And Pelvis |
| 870-879 | Open Wound Of Head, Neck, And Trunk |
| 900-904 | Injury to blood vessels |
| 925-929 | Crushing injury |
| 958 | Certain early complications of Trauma |
| 959.12 | Injury of the abdomen |
| 959.01 | Head injury, unspecified |
| 959.09 | Injury of face and neck |
| 959.9 | Unspecified site injury |
