# Supplementary material for: Modeling the potential impact on the US blood supply of transfusing critically ill patients with fresher stored red blood cells
Source: PLoS One. 2017 Mar 20;12(3):e0174033. doi: 10.1371/journal.pone.0174033 (PMC5358863; doi:10.1371/journal.pone.0174033)
Supplement: S5 Table — (DOCX) [file pone.0174033.s012.docx]

**S5 Table. ICD-9-CM Procedure codes used to identify cardiac patients from the CMS data.**

| **ICD-9-CM Procedure codes** | **Operation description** |
| --- | --- |
| 35 | Operations On Valves And Septa Of Heart |
| 36 | Operations On Vessels Of Heart |
| 37 | Other Operations On Heart And Pericardium |
| 38 | Incision, Excision, And Occlusion Of Vessels |
| 39 | Other Operations On Vessels |
