# Supplementary material for: Modeling the potential impact on the US blood supply of transfusing critically ill patients with fresher stored red blood cells
Source: PLoS One. 2017 Mar 20;12(3):e0174033. doi: 10.1371/journal.pone.0174033 (PMC5358863; doi:10.1371/journal.pone.0174033)
Supplement: S6 Table — Scenario illustration, group description, and allocation method used for each scenario are also presented. (DOCX) [file pone.0174033.s013.docx]

**S6 Table. Percentage of total RBC units transfused and reported by the CMS database for the period 2007-2012 by type of scenario. Scenario illustration, group description, and allocation method used for each scenario are also presented.**

| **Type of scenario** | **Scenario illustration** | **Group Description** | **Percentage of total RBC units transfused during 2007-2012** | **Allocation Method*** |
| --- | --- | --- | --- | --- |
| **ICU ∪ CCU ^†^** ^‡ §^ | 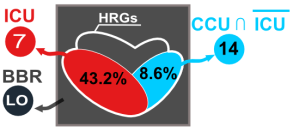 | Any ICU + non-overlapping CCU patients. | ICU 43.2%, CCU 8.6%  ICU ∪ CCU = 51.8% | TM7  TM14 |
|  |  | BBR | 48.2% | LO |
| **CS ∪ CCU ^†^** | 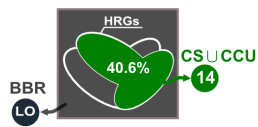 | Any CS + non-overlapping CCU patients. | CS 33.2%, CCU 6.8%  CS ∪ CCU = 40% | TM14  TM14 |
|  |  | BBR | 60% | LO |
| **CS ∪ CCU ∪ ICU ^†^** ^‡§^ | 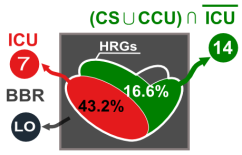 | Any ICU + non-overlapping CS + non-overlapping CCU patients. | ICU 43.2% | TM7 |
|  |  |  | CS ∪ CCU = 16.4%  **CS ∪ CCU ∪ ICU** = 59.9% | TM14 |
|  |  | BBR | 40.1% | LO |

*Allocation methods: LO = Likely Oldest allocation method, Simonetti et al. 2014^1^. TM7, TM14 and TM28 = ‘Threshold Method’, with mean age of RBCs ≤ 7 days, ≤ 14 days and ≤ 28 days, respectively. The ‘threshold’ mean age of RBCs were assumed using recommendations from literature ^4,7^.

**^†^** ∪ denotes union of two or more patient groups (HRGs).

^‡^ ⋂ denotes intersection between two patient groups (HRGs).

^§^ The bar above a patient group, i.e. $\bar{ICU}$, denotes the complement of this group.

ICU=Intensive Care Unit, CS=Cardiac Surgery, CCU=Coronary Care Unit, TR=Trauma and BBR=Baseline Blood Recipient.
